# Regulation of the Fructose Transporter Gene *Slc2a5* Expression by Glucose in Cultured Microglial Cells

**DOI:** 10.3390/ijms222312668

**Published:** 2021-11-23

**Authors:** Tooru M. Mizuno, Pei San Lew, Gursagar Jhanji

**Affiliations:** Division of Endocrinology and Metabolic Diseases, Department of Physiology & Pathophysiology, Max Rady College of Medicine, Rady Faculty of Health Sciences, University of Manitoba, Winnipeg, MB R3E 0J9, Canada; Peisan.Lew@umanitoba.ca (P.S.L.); jhanjig@myumanitoba.ca (G.J.)

**Keywords:** microglia, nutrient, glucose, fructose, obesity

## Abstract

Microglia play a role in the regulation of metabolism and pathogenesis of obesity. Microglial activity is altered in response to changes in diet and the body’s metabolic state. Solute carrier family 2 member 5 (*Slc2a5*) that encodes glucose transporter 5 (GLUT5) is a fructose transporter primarily expressed in microglia within the central nervous system. However, little is known about the nutritional regulation of *Slc2a5* expression in microglia and its role in the regulation of metabolism. The present study aimed to address the hypothesis that nutrients affect microglial activity by altering the expression of glucose transporter genes. Murine microglial cell line SIM-A9 cells and primary microglia from mouse brain were exposed to different concentrations of glucose and levels of microglial activation markers and glucose transporter genes were measured. High concentration of glucose increased levels of the immediate-early gene product c-Fos, a marker of cell activation, *Slc2a5* mRNA, and pro-inflammatory cytokine genes in microglial cells in a time-dependent manner, while fructose failed to cause these changes. Glucose-induced changes in pro-inflammatory gene expression were partially attenuated in SIM-A9 cells treated with the GLUT5 inhibitor. These findings suggest that an increase in local glucose availability leads to the activation of microglia by controlling their carbohydrate sensing mechanism through both GLUT5-dependent and –independent mechanisms.

## 1. Introduction

Microglia are resident macrophages of the central nervous system (CNS) and comprise 0.5–16.6% of the overall cells in the human brain under a healthy CNS environment [1]. As innate immune cells, microglia can initiate immune protection and are responsible for the maintenance of a healthy microenvironment in the brain. Microglia are also involved in synaptic formation/maturation, phagocytic debris removal, and control of neuronal activity. In the absence of CNS insult, microglia continuously move around and scavenge the CNS parenchyma using fine processes with multiple branches. In response to a CNS insult, microglial cells are transformed from their surveillance state to an activated form that displays inflammatory and phagocytic features.

The CNS regulates whole-body metabolism by integrating peripheral metabolic signals. Although specific populations of neurons such as hypothalamic melanocortinergic neurons play a key role in the regulation of metabolism, studies have demonstrated that cell types other than the neuron, such as glial cells also participate in the regulation of metabolism, and abnormalities in these non-neuronal cells contribute to the pathogenesis of obesity and diabetes [2]. Obesity is associated with low-grade inflammation in peripheral metabolic tissues such as adipose tissue and liver. The hypercaloric challenge promotes inflammatory-like responses not only in peripheral tissues but also in the brain, especially in the hypothalamus. High-fat diet (HFD) feeding promotes rapid induction of pro-inflammatory cytokines and inflammatory responsive proteins in the hypothalamus prior to the development of overt obesity [3,4]. These changes are accompanied by increased activation of c-Jun N-terminal kinase (JNK) and IκB kinase (IKK)/nuclear factor-kappa B (NF-κB) that interferes with metabolic actions of insulin and leptin in the hypothalamus, resulting in insulin or leptin resistance, respectively [3,5]. The consumption of HFD leads to an accumulation of activated microglia and increased expression of pro-inflammatory genes in hypothalamic microglia [6]. Additionally, it was found that a HFD feeding stimulates the recruitment of peripheral myeloid cells into the CNS, leading to an increase in the number of the microglia in the hypothalamus [7]. These findings suggest that acute activation of hypothalamic microglia is an adaptive response to hypercaloric challenge and reduces food intake, while prolonged activation of microglia and hypothalamic inflammation may contribute to HFD-induced obesity. Consistent with this assumption, a study had shown that microglial depletion or blocking the microglial inflammatory pathway attenuates HFD-induced hyperphagia and weight gain in mice. Conversely, forcing microglial activation is sufficient to stimulate microgliosis in the hypothalamus, which subsequently causes an increase in food intake and weight gain in standard chow-fed mice [8]. Together, these findings emphasize the importance of the inflammatory activation state of microglia in the regulation of food intake and its role in the pathogenesis of HFD-induced obesity.

HFD-induced microglial activation and inflammatory response in the hypothalamus may not be the consequence of weight gain, as mouse models of monogenic obesity do not show reactive microglia in any brain region when they are placed on a standard chow diet [9]. Although microglial depletion does not alter food intake when mice are fed with a regular rodent chow diet, it reduces chow intake when mice receive gavage feeding with a saturated fat solution in addition to the regular rodent chow [6]. Thus, dietary factors may affect microglial activity independent of changes in body weight and prolonged activation of microglia may interfere with nutrient sensing by the hypothalamus, resulting in an increased food intake.

Glucose is the primary energy substrate of not only neurons but also microglia [10]. Glucose sensing by hypothalamic neurons, astrocytes, and tanycytes is an important component of CNS regulation of whole-body metabolism [11,12]. Previous studies have demonstrated that central administration of the pro-inflammatory cytokine interleukin-1 (IL-1) alpha or beta reduces food intake in rodents [13,14]. Levels of interleukin 1 alpha (*Il1a*) and beta (*Il1b*) mRNA are increased in cultured mouse hypothalamic explants in the presence of high glucose compared to low glucose [15]. Glucose induces activated ameboid morphology and stimulates phosphorylation of JNK alongside the release of pro-inflammatory cytokines in microglial cells [16,17,18,19,20]. In humans, hyperglycemia is associated with increased microglial activation in patients with a critical illness [21]. Interestingly, glucose-induced feeding suppression is attenuated in IL-1 receptor-deficient mice [15]. These findings suggest the possibility that hypothalamic microglia detect changes in glucose availability and mediate the acute anorexigenic effect of glucose.

Glucose transporter 5 (GLUT5) is the hexose transporter with a much greater affinity to fructose compared to glucose and is primarily expressed in microglia within the CNS [22,23]. Fructose feeding increases the expression of GLUT5 in the brain and results in an increased activity of microglia [24,25]. Fructose induces the activation of the NF-κB pathway and increases the expression and release of pro-inflammatory cytokines by microglial cells [26,27]. These findings suggest that glucose and/or fructose might be nutrient factors that regulate microglial activity via GLUT5 and that the glucose-induced microglial inflammatory response mediates the anorexigenic action of glucose. However, there is little information on how the increased availability of simple sugars affects microglial activity and the expression of glucose transporters.

A number of in vitro cell models have been established to study microglial function [28]. Although primary microglia culture is reflective of the in vivo experiment and therefore a preferred model to study the physiology of microglia, it has its disadvantages such as being time-consuming, costly in its preparation, difficulty in obtaining satisfactory purity and yield and having inter-animal variations. To overcome these disadvantages, cell lines have been established as an alternate cell model. There are a variety of microglial cell lines derived from the mouse brain and most of these lines such as the widely used BV-2 and N-9 cells were immortalized by viral transformation with oncogenes that may affect the normal function of microglia [28]. In contrast to these virally transformed immortalized cell lines, SIM-A9 is a spontaneously immortalized microglial cell line that was established from mouse cortical tissues [29]. SIM-A9 cells display microglia-like morphologies and express microglial markers ionized calcium-binding adapter molecule 1 (Iba1) and CD68, while they are negative for astrocyte and neuronal markers. SIM-A9 cells exhibit phagocytic activity and increased expression and secretion of pro-inflammatory cytokines in response to inflammatory stimuli such as lipopolysaccharide (LPS) [29,30]. BV-2 cells show a reduced response to external stimuli after a number of passages, while SIM-A9 cells retain microglial phenotypes at least up to 40 passages [29]. These findings suggest that the SIM-A9 cells may serve as a reliable alternative model of primary microglia and virally transformed microglial cell lines to investigate the microglial function and gene expression.

The present study aimed to address the hypothesis that nutrients affect microglial activity and expression of glucose transporter genes by investigating the effect of glucose on the levels of microglial activation markers and glucose transporter genes expression in mouse primary microglia and SIM-A9 cells.

## 2. Results

### 2.1. Expression of Glucose Transporter Genes in Mouse Primary Microglia and Mouse Microglial Cell Line SIM-A9 Cells

**Figure 1:**
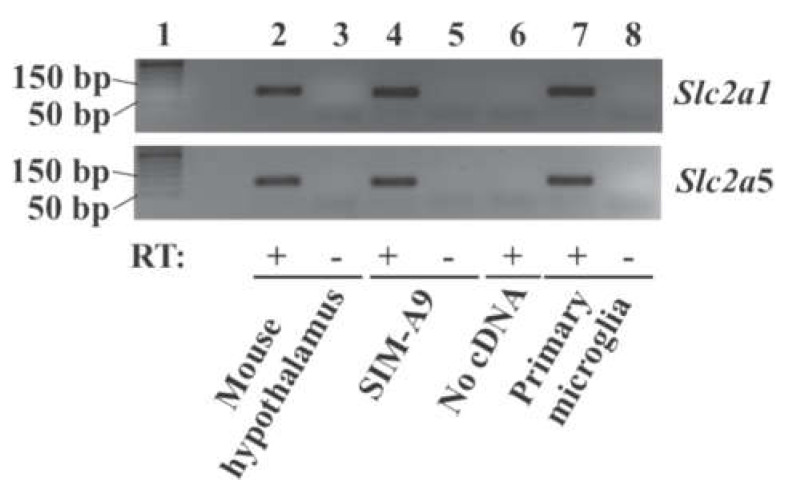
Expression of the glucose transporter gene *Slc2a1* and *Slc2a5* in microglial cells. Total RNA was extracted from microglial SIM-A9 cells, mouse primary microglia, and mouse hypothalamus (positive control), treated with DNase I, and converted to cDNA. *Slc2a1* and *Slc2a5* were amplified by RT-PCR. PCR products (94 bp for *Slc2a1* and 100 bp for *Slc2a5*) were analyzed by gel electrophoresis. Lane 1: 50 bp DNA ladder; lane 2, 3: mouse hypothalamus; lane 4, 5: SIM-A9 cells; lane 6: no RNA/cDNA; lane 7, 8: mouse primary microglia. RT: Reverse transcriptase.

To determine whether genes encoding GLUT1, GLUT2, and GLUT5 are expressed in our mouse primary microglial cells and SIM-A9 cells, expression of solute carrier family 2 member 1 (*Slc2a1*), *Slc2a2*, and *Slc2a5* mRNA was checked in these cells. RT-PCR showed that *Slc2a1* and *Slc2a5* mRNA were present in mouse hypothalamus (positive control), primary microglial cells from mouse brain, and SIM-A9 cells (Figure 1). RT-PCR successfully amplified an expected fragment from mouse liver RNA (positive control) using *Slc2a2*-specific primers, while it failed to amplify the *Slc2a2* fragment from primary mouse microglia and SIM-A9 cells in the present study (data not shown).

### 2.2. Effect of Glucose on the Activity of Mouse Primary Microglia

**Figure 2:**
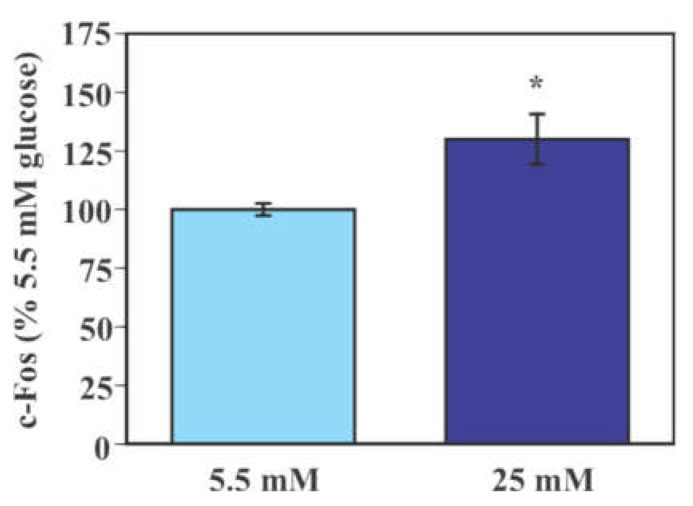
Glucose-induced activation of mouse primary microglia. Cells were incubated in a culture medium containing either low (5.5 mM) or high (25 mM) glucose for 40 min. Levels of c-Fos were measured by Western blot analysis and were normalized to total protein from stain-free blot for each sample. Values in the control group (low glucose) were set to 100%. Data are means ± S.E.M. (*n* = 6/group). *: *p* < 0.05 by Student’s *t*-test.

Levels of the immediate early gene product c-Fos were significantly higher in microglial cells exposed to 25 mM glucose compared to those exposed to 5.5 mM glucose (30.0% increase, *p* < 0.05, Figure 2).

### 2.3. Effect of Glucose on the Expression of Genes Encoding Glucose Transporters and Pro-Inflammatory Cytokines in Mouse Primary Microglia

**Figure 3:**
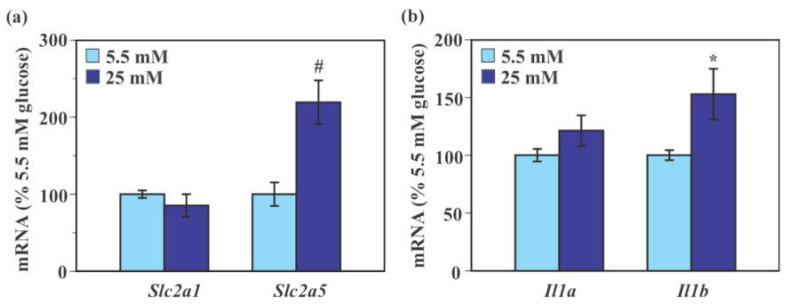
Effect of glucose on the expression of glucose transporter and pro-inflammatory genes in mouse primary microglia. Cells were incubated in a culture medium containing either low (5.5 mM) or high (25 mM) glucose for 40 min. Levels of *Slc2a1*, *Slc2a5* (**a**), *Il1a,* and *Il1b* (**b**) mRNA were measured by real-time PCR. Values in the control group (low glucose) were set to 100%. Data are means ± S.E.M. (*n* = 8–9/group). *: *p* < 0.05, ^#^: *p* < 0.005 by Student’s *t*-test.

Exposure to 25 mM glucose caused a significant 119.3% increase in *Slc2a5* mRNA level (*p* < 0.005) compared to 5.5 mM glucose without causing a significant change in *Slc2a1* mRNA level in mouse primary microglia (*p* = 0.33, Figure 3a). Levels of *Il1a* mRNA were indistinguishable between 5.5 mM and 25 mM glucose (*p* = 0.14). Levels of *Il1b* mRNA were significantly increased in 25 mM glucose-treated cells (52.9% increase, *p* < 0.05) compared to 5.5 mM glucose-treated cells (Figure 3b).

### 2.4. Effect of Glucose on the Activity of SIM-A9 Cells

**Figure 4:**
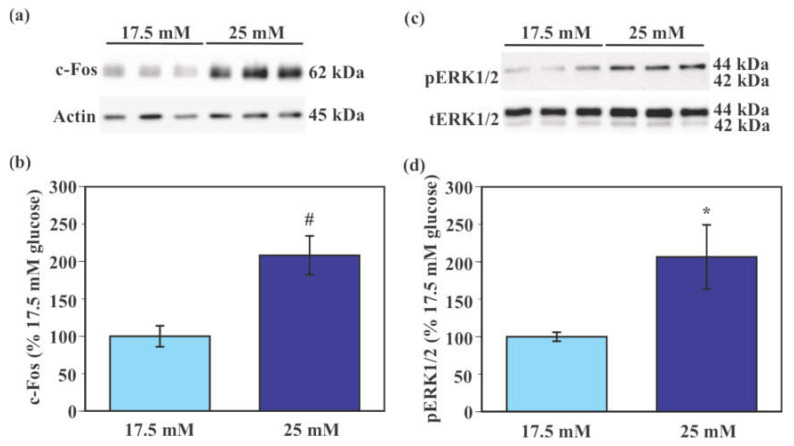
Glucose-induced activation of murine microglial SIM-A9 cells. Cells were incubated in a culture medium containing either 17.5 mM or 25 mM glucose for 40 min. Levels of c-Fos, pERK1/2, total ERK1/2, and pan-actin were measured by Western blot analysis and were normalized to total protein from stain-free blot for each sample. Levels of pERK1/2 were normalized to levels of total ERK1/2. Representative image of Western blot of c-Fos, pan-actin (**a**), pERK1/2, and total ERK1/2 (**c**). Values in the control group (17.5 mM glucose) were set to 100% (**b**,**d**). Data are means ± S.E.M. (*n* = 10–12/group). *: *p* < 0.05, ^#^: *p* < 0.005 by Student’s *t*-test.

Expression of c-Fos protein was significantly higher in SIM-A9 cells exposed to 25 mM glucose compared to those exposed to 17.5 mM glucose (108.2% increase, *p* < 0.005, Figure 4a,b). Levels of phosphorylated extracellular signal-regulated kinase 1/2 (pERK1/2) were significantly higher in SIM-A9 cells exposed to 25 mM glucose compared to those exposed to 17.5 mM glucose (106.4% increase, *p* < 0.05, Figure 4c,d).

### 2.5. Effect of Glucose on the Expression of Genes Encoding Glucose Transporters and Pro-Inflammatory Cytokines in SIM-A9 Cells

**Figure 5:**
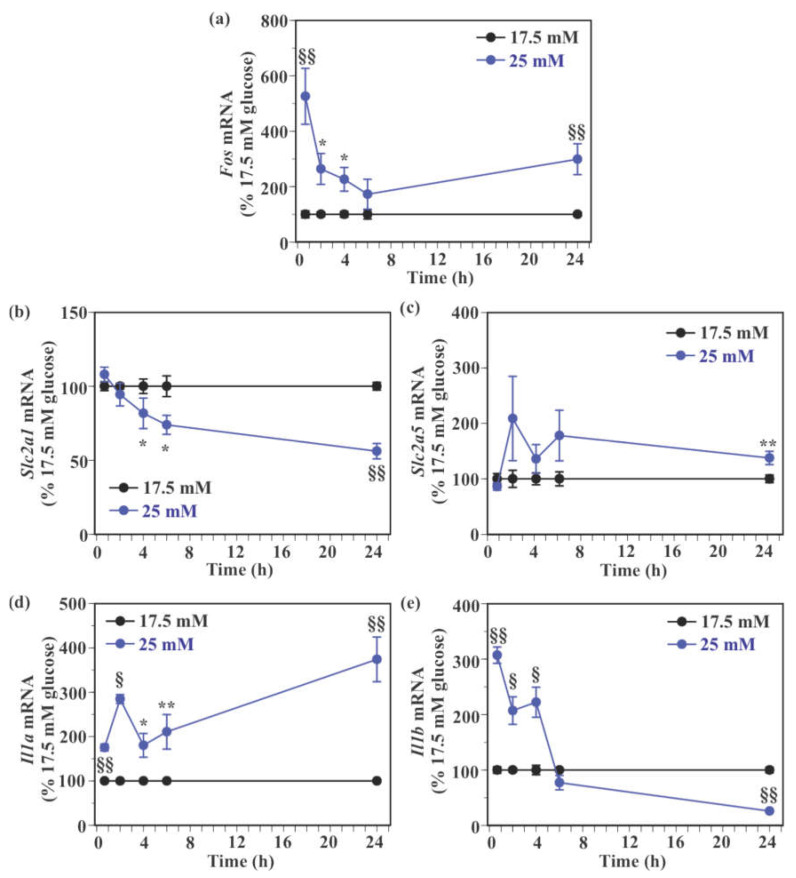
Effect of glucose on the expression of the immediate early gene, glucose transporter, and pro-inflammatory genes in murine microglial SIM-A9 cells. Cells were incubated in a culture medium containing either 17.5 mM or 25 mM glucose for 40 min, 2, 4, 6, or 24 h. Levels of *Fos* (**a**), *Slc2a1* (**b**), *Slc2a5* (**c**), *Il1a* (**d**), and *Il1b* (**e**) mRNA were measured by real-time PCR. Values in the control group (17.5 mM glucose) were set to 100% at each time point. Data are means ± S.E.M. (*n* = 11–12/group). *: *p* < 0.05, **: *p* < 0.01, ^§^: *p* < 0.0005, ^§§^: *p* < 0.0001 vs. 17 mM glucose at each time point by Student’s *t*-test or Wilcoxon test.

Similar to c-Fos protein levels, *Fos* mRNA levels were significantly higher in SIM-A9 cells exposed to 25 mM glucose for 40 min (426.1% increase), 2 h (163.9% increase), 4 h (126.4% increase), and 24 h (199.0% increase) compared to those exposed to 17.5 mM glucose without a significant difference at 6 h (Figure 5a). There was no significant difference in *Slc2a1* mRNA level between 17.5 mM and 25 mM glucose at 40 min and 2 h (Figure 5b), whereas *Slc2a1* mRNA levels were significantly reduced by 25 mM glucose at 4 h (18.3% reduction), 6 h (26.1% reduction), and 24 h (43.9% reduction) compared to 17.5 mM glucose (Figure 5b). Levels of *Slc2a5* mRNA were significantly increased 24 h after exposure to 25 mM glucose compared to 17.5 mM glucose (37.7% increase, Figure 5c). High glucose caused a non-significant increase in *Slc2a5* mRNA expression at 2 h (108.8% increase), 4 h (35.9% increase), and 6 h (78.1% increase), while it did not increase *Slc2a5* mRNA levels at 40 min (Figure 5c). Exposure to 25 mM glucose caused a significant increase in *Il1a* mRNA level compared to 17.5 mM glucose at all examined time points (75.3–274.1% increase, Figure 5d). Exposure to 25 mM glucose significantly increased *Il1b* mRNA levels at 40 min (207.1% increase), 2 h (107.2% increase), and 4 h (122.3% increase) compared to 17.5 mM glucose, while it caused a significant reduction in *Il1b* mRNA expression at 24 h (74.0% reduction) without a significant change at 6 h (Figure 5e).

### 2.6. Effect of Fructose on the Expression of Genes Encoding Glucose Transporters and Pro-Inflammatory Cytokines in SIM-A9 Cells

**Figure 6:**
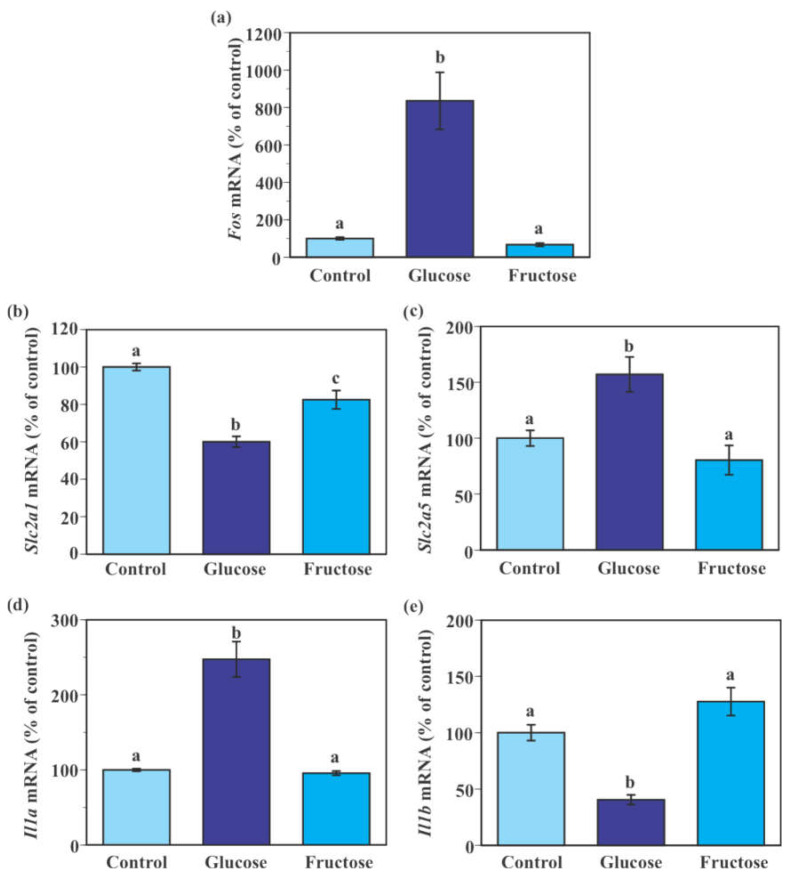
Effect of fructose on the expression of glucose transporter and pro-inflammatory genes in murine microglial SIM-A9 cells. Cells were incubated in a culture medium containing 17.5 mM glucose, 25 mM glucose (7.5 mM glucose + 17.5 mM glucose), or 7.5 mM fructose + 17.5 mM glucose for 24 h. Levels of *Fos* (**a**), *Slc2a1* (**b**), *Slc2a5* (**c**), *Il1a* (**d**), and *Il1b* (**e**) mRNA were measured by real-time PCR. Values in the control group (17.5 mM glucose) were set to 100%. Data are means ± S.E.M. (*n* = 10–14/group). Groups that do not share a common letter are statistically different (*p* < 0.05 by Tukey–Kramer test).

Consistent with the results of the aforementioned time-course experiment (Figure 5), 24-h incubation with 25 mM glucose (an addition of 7.5 mM glucose to the maintenance 17.5 mM glucose) for 24 h caused significant increases in *Fos*, *Slc2a5*, and *Il1a* mRNA expression and significant reductions in *Slc2a1* and *Il1b* mRNA expression in SIM-A9 cells compared to the control 17.5 mM glucose (Figure 6a–e). Incubation of cells with fructose (an addition of 7.5 mM fructose to the maintenance 17.5 mM glucose) significantly reduced *Slc2a1* mRNA levels by 17.5% compared to the control 17.5 mM glucose (Figure 6b). Levels of *Fos*, *Slc2a5*, *Il1a,* and *Il1b* mRNA were indistinguishable between control and fructose-treated groups (Figure 6a,c–e).

### 2.7. Expression of Slc2a5 mRNA in Activated SIM-A9 Cells

To determine whether an increased activity of microglia is associated with increased *Slc2a5* mRNA expression, SIM-A9 cells were treated with lipopolysaccharide (LPS). LPS treatment for 4 h caused a significant increase in *Il1a*, *Il1b*, and *Slc2a5* mRNA levels compared to the control (Table 1).

### 2.8. Effect of GLUT5 Inhibition on the Expression of Pro-Inflammatory Cytokine Genes in SIM-A9 Cells

**Figure 7:**
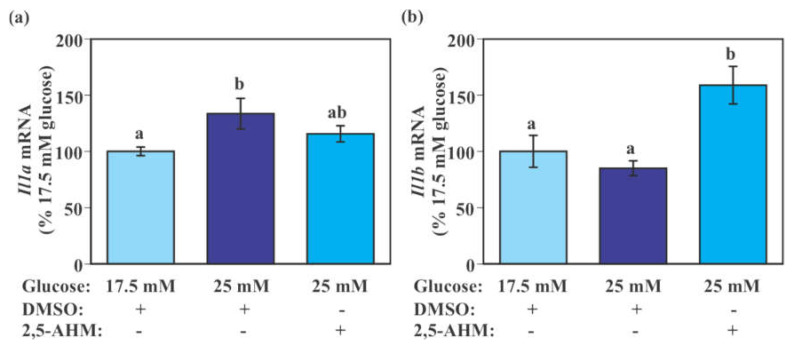
Effect of GLUT5 inhibitor on the expression of pro-inflammatory genes in murine microglial SIM-A9 cells. Cells were incubated in a culture medium containing 17.5 mM glucose with DMSO (vehicle control) or 25 mM glucose in the presence of 2,5-AHM (2 mM) or DMSO for 24 h. Levels *Il1a* (**a**) and *Il1b* (**b**) mRNA were measured by real-time PCR. Values in the control group (17.5 mM glucose + DMSO) were set to 100%. Data are means ± S.E.M. (*n* = 9–10/group). Groups that do not share a common letter are statistically different (*p* < 0.05 by Tukey–Kramer test).

SIM-A9 cells exposed to 25 mM glucose for 24 h had significantly higher *Il1a* mRNA levels compared to those exposed to 17.5 mM glucose (*p* = 0.0389 by Tukey–Kramer test). Although the difference was not statistically significant, levels of *Il1a* mRNA in SIM-A9 cells exposed to 25 mM glucose with 2,5-AHM treatment were intermediate between those of 17.5 mM glucose without 2,5-AHM treatment and 25 mM glucose without 2,5-AHM treatment (Figure 7a). There was a trend of reduced *Il1b* mRNA by the 24-h incubation with 25 mM glucose; however, this was not statistically significant in this experiment. 2,5-AHM treatment significantly increased *Il1b* mRNA levels compared to the control DMSO treatment when cells were exposed to 25 mM glucose (*p* = 0.0013 by Tukey–Kramer test, Figure 7b).

## 3. Discussion

Hypothalamic nutrient sensing plays a pivotal role in the regulation of whole-body metabolism. The hypothalamus is a ventral forebrain structure surrounded by the third ventricle. Both the location and structure of the hypothalamus allow subsets of the hypothalamic neurons to sense circulating nutrients and hormones. It is not surprising that microglial cells within this brain region also possess a similar ability to sense changes in circulating levels of nutrients and hormones. Consumption of a diet rich in saturated fats as well as intracerebroventricular (i.c.v.) administration of saturated fatty acids increases the activity of microglia and stimulates inflammatory responses [3,4,31]. Similarly, a high glucose/high cholesterol diet promotes the development of obesity-like and diabetes-like phenotypes in zebrafish, accompanied by microglial activation and increased expression of pro-inflammatory cytokine genes [32]. Activation of microglia is observed in hyperglycemic patients, but it is not found in individuals with normoglycemia, suggesting that elevated levels of glucose increase microglial activity [21]. To support this assumption, in vitro studies have provided evidence for glucose regulation of microglial activity. An elevated glucose concentration induces a morphological change from resting ramified microglia to activated ameboid microglia and stimulates expression and release of pro-inflammatory cytokines by primary microglia, BV-2 cells, and HMC3 cells [16,17,18,19,20,33,34]. The present study demonstrated that both the primary mouse microglia and SIM-A9 cells responded to increased concentrations of glucose as represented by increased expressions of c-Fos and pERK1/2. Increased glucose concentration also caused a time-dependent change in the expression of pro-inflammatory genes *Il1a* and *Il1b*. Consistent with previous studies reporting different patterns of *Il1a* and *Il1b* mRNA expression following pro-inflammatory stimulus in cells such as microglia [35,36], levels of *Il1a* mRNA remained at a high level up to 24 h after exposure to high glucose in SIM-A9 cells, while levels of *Il1b* mRNA reached a peak at 40 min and were reduced at 24 h in the present study. These data suggest that microglia produce an inflammatory response through precise temporal coordination of pro-inflammatory gene expression following exposure to high glucose. These findings provide evidence about the ability of microglia to respond to a change in local glucose availability and increase its activity and inflammatory response as part of the CNS glucose sensing system. It is further suggested that SIM-A9 cells can serve as an additional model of microglia to study the nutritional regulation of microglial gene expression and function.

It should be noted that previous studies, as well as the present study, examined the effect of glucose on the microglial activity at extremely high concentrations (16.7–35 mM) [16,17,18,20]. These concentrations do not represent extracellular glucose concentration in the brain under normal condition (0.7–2.5 mM) [37,38]. However, these high glucose concentrations may represent those that were seen following an i.c.v. injection of glucose at a dose that can produce an acute reduction of food intake in mice. For example, an i.c.v. administration of 100–400 μg glucose causes changes in levels of feeding-related molecules in the hypothalamus and reduces food intake in mice [39,40]. Since the volume of the cerebrospinal fluid (CSF) ranges from 35 to 40 μL in mice, CSF glucose concentration may reach 14–64 mM after the i.c.v. glucose injection at these doses [41,42]. Additionally, not only high glucose but hypoglycemia also induces microglia activation and expression of pro-inflammatory cytokines genes in the hypothalamus [43]. Despite that, insulin-induced hypoglycemia and even glucose deprivation did not cause a change in microglia morphology from ramified to ameboid shape [44,45]. Thus, the effect of glucose on microglia may vary depending on glucose concentrations to be compared. It is important to investigate the microglial response to changes in glucose concentration within a more physiological range in future studies.

The possible mechanism that mediates glucose-induced microglial activation and inflammatory response hereby remains the topic of curiosity. Previous studies have suggested that microglial activation is mediated via specific glucose transporters. GLUT1-encoding *Slc2a1* mRNA is expressed at the highest level among other glucose transporter genes in microglia. LPS and interferon-gamma (INFγ)-induced microglial activation is associated with increased *Slc2a1* mRNA and GLUT1 protein expression, glucose uptake, and glycolysis in BV-2 and B6M7 microglia without significant changes in expression of other glucose transporter genes and proteins. LPS and INFγ-induced activation of microglia were abolished by treatment with a specific GLUT1 inhibitor [46]. GLUT2 is critical for hypothalamic glucose sensing and glucose-induced feeding suppression [47]. The transition from low to high glucose increases proliferation, which coincides with increased GLUT2, but not GLUT1 and GLUT5 expression in BV-2 cells. These changes are accompanied by increased secretion of pro-inflammatory cytokines and up-regulation of microglial activation markers such as pERK1/2. Additionally, the effect of high glucose on cell proliferation is negated by GLUT2 knockdown [20]. These data suggest that microglia facilitate glucose uptake/utilization and increase activity via GLUT1 and GLUT2. Contrary to these findings, high glucose concentrations did not increase *Slc2a1* mRNA expression in primary mouse microglia (no change) and SIM-A9 cells (reduction), while *Slc2a2* mRNA was not detected in these cells in the present study. Although the exact reason for this discrepancy is unknown, different microglial cell models may exhibit different phenotypes. Additionally, the present study measured GLUT-encoding mRNA levels, while a study by Hsieh et al. measured GLUT protein levels [20]. Interestingly, the present study shows that LPS treatment increased the expression of pro-inflammatory genes as well as *Slc2a5* mRNA in SIM-A9 cells (Table 1). We also found that a glucose-induced increase in *Il1a* mRNA expression was partially blocked by GLUT5 inhibitor treatment in SIM-A9 cells (Figure 7a). This is in agreement with previous reports that although fructose is the main inducer of *SLC2A5* gene expression, glucose also increases *SLC2A5* promoter activity, mRNA expression, and GLUT5 protein expression in the human colon cancer cell line [48,49]. Collectively, these data support the possibility that increased local glucose level causes activation of microglia and changes in pro-inflammatory gene expression, and that these changes are partially mediated by a GLUT5-dependent mechanism. Follow-up studies are necessary to further determine the contribution of GLUT1, 2, and 5 to glucose-induced activation of microglia and inflammatory response.

Little is known about the role and regulation of GLUT5 in microglia. It was previously reported that fructose feeding in rats increased the expression of *Slc2a5* mRNA and GLUT5 protein in the brain [24]. Excessive fructose intake results in increased activity of microglia [25]. Moreover, fructose induces the activation of the NF-κB pathway and increases expression and secretion of pro-inflammatory cytokines by microglial BV-2 cells [26,27]. These findings raise the possibility that the conversion of glucose to fructose in the brain may be required for glucose-induced increase in *Slc2a5* mRNA expression and microglial activation. The polyol pathway of glucose metabolism becomes active when intracellular glucose levels are elevated. Approximately 30% of glucose can be converted to fructose via the polyol pathway under hyperglycemic conditions [50]. A recent study demonstrated that brain glucose levels rise during a hyperglycemic clamp in healthy adult individuals. Intriguingly, intracerebral fructose levels were also increased, and these changes were associated with changes in brain glucose but not plasma fructose levels [51]. These data provide evidence for the endogenous conversion of glucose to fructose within the brain. However, the addition of fructose (7.5 mM) to the maintenance culture medium containing 17.5 mM glucose failed to increase levels of *Slc2a5* mRNA and did not alter the expression of *Fos*, *Il1a*, and *Il1b* mRNA in SIM-A9 cells in the present study. This is in marked contrast to the pronounced effect of glucose (25 mM glucose by adding 7.5 mM glucose to the maintenance 17.5 mM glucose) on the expression of these genes. Taken together, when exposed to high glucose concentrations, microglia increase *Slc2a5* gene expression by responding to elevated glucose, but not to elevated fructose. Thus, glucose may directly stimulate pro-inflammatory genes expression in microglia, while fructose (or conversion of glucose to fructose) may have a minimal contribution to glucose-induced changes in pro-inflammatory genes expression. Whether conversion of glucose to fructose is required for glucose-induced activation of microglia awaits further study.

In contrast to the anorexigenic effect of glucose, central administration of fructose increases food intake in rodents [40,52]. Fructose is mainly metabolized in the small intestine after intestinal absorption, resulting in exceedingly low circulating levels of fructose under normal conditions. When fructose intake exceeds the fructose clearing capacity of the small intestine, other tissues such as the liver will be exposed to fructose [53]. Circulating fructose level rapidly reaches a peak, enters, and is metabolized by the brain following an intraperitoneal (i.p.) injection of fructose [54]. Peripherally administered fructose is also converted to glucose in the liver, resulting in an increased blood glucose level with a peak that appears after the peak of fructose [40]. These findings suggest that fructose may not have a significant effect on CNS cells including microglia under normal conditions where blood fructose levels are low, but may influence the activity of CNS cells through its direct action and/or interconversion of fructose to glucose when a large amount of fructose is ingested. These findings also suggest a novel idea that microglia have the unique ability to respond to two-opposing monosaccharides, anorexigenic glucose, and orexigenic fructose allowing fine-tuning of microglial function and metabolic regulation under a variety of conditions with different combinations of glucose and fructose levels.

The present study suggests that glucose and/or fructose may be nutrient factors that regulate microglial activity through both GLUT5-dependent and –independent mechanisms. When the local concentration of glucose is elevated, microglia are activated and produce pro-inflammatory cytokines that can produce anorexigenic action. Thus, activated microglia reduce food intake in response to acutely increased local glucose and/or fructose availability, at least partly, via GLUT5 and maintain the normal energy balance of the body. In contrast, glucose and fructose, when overloaded for a prolonged period of time, become deleterious. Constantly elevated local glucose concentration as well as fructose may cause chronic inflammation and impair microglial function, leading to hyperphagia and abnormally increased weight gain. Understanding the precise mechanism by which microglia regulate metabolism in response to changes in glucose and fructose availability may suggest an effective treatment for obesity and associated impairments.

## 4. Materials and Methods

### 4.1. Primary Microglial Culture

The brains were harvested from three neonatal mouse pups on P2-P4 under sterile conditions. Brain tissues were dissected, separated from the meninges under a microscope, rinsed in dissecting buffer (2 mM HEPES, 1X HBSS, 50 U/mL penicillin, and 50 μg/mL streptomycin). The tissue was minced finely and digested with 1X Trypsin dissociation media for 3 min in the CO_2_ incubator at 37 °C. The enzymatic digestion was terminated by the addition of glia growth medium (1X MEM, 11090-099, Gibco by Thermo Fisher Scientific, Waltham, MA, USA) supplemented with 10% fetal bovine serum (FBS, A3160702, Gibco), 2 mM L-glutamine (25030081, Gibco) and streptomycin sulfate (S9137, Sigma-Aldrich, St. Louis, MO, USA). The media were removed carefully by gentle pipetting to not disturb the detached cells. The cell suspension was gently mixed with fresh growth media by pipetting up and down and transferred into a T25 flask containing pre-warmed growth media. They were allowed to grow in a 37 °C incubator with 5% CO_2_/95% O_2_. Cells were inspected at a 4-h time-point for growth. The culture medium was replaced every 2 days to remove cell debris. Between days 7–10, confluent astrocytes were observed and there was significant growth of microglia. Microglia were isolated from the mixed glial cultures by shaking. Media containing floating cells were collected and re-seeded into multi-well culture plates for treatment. One hour later, non-adherent cells were removed by gently using a sterile serological pipette and transferred to a flask containing pre-warmed growth media. The isolated microglia were allowed to equilibrate for 24 h prior to treatments. The purity of the primary microglial culture was confirmed by immunocytochemistry. Almost all the cells were positive for Iba1 and a few cells were positive for glial fibrillary acidic protein (GFAP). Mixed glial cells were passed into a bigger T75 flask once they became dense in the original T25 culture flask at a 1:2 ratio. Phenol red-free TrypLE Express (12604021, Gibco) was used as a dissociation enzyme. Cells from passages 3–9 were used in the present study. To determine the effect of glucose, primary microglial cells were incubated in a culture medium containing 5.5 mM glucose for 24 h followed by exposure to either low (5.5 mM) or high (25 mM) glucose for 40 min. At the end of the incubation period, cells were harvested and used for protein or gene expression analysis. All procedures involving animals were conducted according to the guidelines of the Declaration of Helsinki and approved by the Institutional Animal Care and Use Committee of the University of Manitoba (protocol number 17-044, 10 June 2020).

### 4.2. Culture of SIM-A9 Microglia Cell Line

The immortalized mouse microglial cell line SIM-A9 (ATCC^®^, CRL-3265™) was established from mouse cortical tissues and exhibit key characteristics of microglia [29]. Cells were maintained in a T75 flasks containing DMEM/F-12 (D8900, Sigma-Aldrich, St. Louis, MO, USA) supplemented with 10% FBS (A3160702, Gibco, Waltham, MA, USA), 5% heat-inactivated horse serum (H1138, Sigma-Aldrich, St. Louis, MO, USA), 50 U/mL penicillin, 50 μg/mL streptomycin and 100 μg/mL neomycin (P4083, Sigma-Aldrich, St. Louis, MO, USA) in a 37 °C incubator with 5% CO_2_/95% O_2_. Once a 90% confluency was reached, cells were shaken by hand and seeded into 6-well plates. To prevent wastage or over-aging, cells were passaged at a 1:2 ratio using TrypLE Express dissociation media when not in use. Cells from passages 12–13 were used in the present study.

SIM-A9 cells have been maintained in DMEM/F12 culture medium containing 17.5 mM glucose in previous studies [29,30,55] and other glucose concentrations have never been used. In our pilot study, we examined the effect of transition of glucose concentration from the maintenance 17.5 mM to either 5.5 mM (low) or 25 mM (high) on microglial activation. As we report in the present study, the transition from 17.5 mM to 25 mM glucose caused an increase in c-Fos and pERK1/2 levels in SIM-A9 cells (Figure 4). Interestingly, levels of c-Fos and pERK1/2 were also elevated when glucose concentration was lowered from 17.5 mM to 5.5 mM in SIM-A9 cells (Figure S1). Although these findings are interesting, our intention in the present study was not a direct comparison between primary microglia and SIM-A9 cells. Instead, we sought to study glucose-induced microglial activation. Accordingly, we decided to focus on the transition from 17.5 mM to 25 mM glucose in SIM-A9 cells in the present study.

To determine the effect of glucose, cells in T75 flasks were shaken and seeded into multi-well plates and incubated in a maintenance medium containing 17.5 mM glucose for 24 h before incubation in a culture medium containing either 17.5 mM glucose (control) or 25 mM glucose for 40 min, 2, 4, 6, or 24 h. To determine the effect of fructose, cells were incubated in a maintenance medium containing 17.5 mM glucose for 24 h followed by incubation in a culture medium containing 17.5 mM glucose (control), 25 mM glucose (positive control, addition of 7.5 mM glucose to the maintenance 17.5 mM glucose), or 7.5 mM fructose plus 17.5 mM glucose for 24 h. The 7.5 mM fructose was chosen to keep the molar of simple sugar identical to that of the high glucose treatment group (7.5 mM glucose plus 17.5 mM glucose). To determine the effect of LPS on *Slc2a5* mRNA expression, SIM-A9 cells were incubated in a culture medium containing 17.5 mM glucose for 24 h and treated with LPS (2.5 ng/mL, L5886, Sigma-Aldrich) for 4 h. To determine the effect of GLUT5 inhibition, cells were incubated in a maintenance medium (17.5 mM glucose) for 24 h before incubation in a culture medium containing either 17.5 or 25 mM glucose with or without 2 mM 2,5-anhydro-D-mannitol (2,5-AHM, 21673, Cayman Chemical, Ann Arbor, MI, USA) for 24 h. Dimethyl sulfoxide (DMSO) was used as a vehicle control. This concentration of 2,5-AHM has been reported to be effective in blocking cellular response to fructose in cell culture experiments [56,57]. At the end of the incubation period, cells were harvested for protein or gene expression analysis.

### 4.3. Western Blot Analysis

Iba1 is a frequently used marker of activated microglia. However, a previous study as well as our pilot study failed to show a robust change in Iba1 expression in SIM-A9 cells following LPS treatment [29]. The immediate early gene product c-Fos has been widely used as an indirect marker of cell (neurons as well as other cell types) activation in response to nutrient and hormonal signals such as glucose [58,59]. Intriguingly, a recent study demonstrated that c-Fos expression was increased in response to LPS/ATP treatment in HMC3 microglial cells [60]. Another study reported that a high concentration of glucose increases phosphorylation of ERK1/2 in BV-2 microglia [20]. Based on these findings, we decided to measure levels of c-Fos and pERK1/2 as markers of glucose-induced microglial activation in the present study.

Cells were lysed in protein lysis buffer (50 mM HEPES, 150 mM NaCl, 10% glycerol, and 1% Triton X-100) supplemented with an EDTA-free proteinase inhibitor cocktail (Roche Diagnostics, Indianapolis, IN, USA). Total protein concentrations were determined by Bradford assay using bovine serum albumin as a standard. Proteins (10–25 μg) were separated on 10% stain-free gels (Bio-Rad Laboratories, Hercules, CA, USA) at 175 V or 250 V. They were then transferred to pre-wet low fluorescent polyvinylidene difluoride (PVDF) membranes (Bio-Rad Laboratories) at 100 V for 1–1.5 h. The blots were rinsed with Tris-buffered saline (TBS) and total proteins transferred were imaged using a stain-free technology using ChemiDoc MP imager (Bio-Rad Laboratories). After blocking with 5% non-fat milk in 1X TBS with 0.05% Tween 20 (TBST) for 0.5–1 h at room temperature, membranes were incubated overnight at 4 °C in primary antibodies for c-Fos (226003, Synaptic Systems, Göttingen, Germany, 1:200), pERK1/2 (Thr202/Tyr204, 9101, Cell Signaling Technology, Danvers, MA, USA, 1:500) or ERK1/2 (9102, Cell Signaling Technology, 1:500). For loading control, membranes were incubated with pan-actin antibody (4968, Cell Signaling Technology, 1:1,000) for 1 h at room temperature. After washing in TBST, membranes were incubated with goat anti-rabbit horseradish peroxidase (HRP)-conjugated secondary antibody (111-035-03, Jacksons ImmunoResearch Laboratories, West Grove, PA, USA) at 1:5,000 dilution in blocking buffer for 30-60 min at room temperature. Membranes were first incubated with either c-Fos or pERK1/2 before being stripped for 30 min using glycine stripping buffer (pH 2.2) prior to incubation with the other antibody. Protein bands were visualized using an enhanced chemiluminescence (ECL) and imaged using the ChemiDoc MP imager. The intensity of the bands was quantified using the ImageLab software ver. 6 (Bio-Rad Laboratories). In a pilot study, we found that the activation status of microglia (e.g., stimulation by LPS or metabolic hormones) influences actin levels. Therefore, the intensity of c-Fos was normalized to total protein from stain-free blot for each sample. The intensity of pERK1/2 was normalized to total ERK1/2.

### 4.4. RNA Analysis

To determine whether *Slc2a1*, *Slc2a2*, and *Slc2a5* mRNA are expressed in SIM-A9 and primary microglia cells, total RNA was extracted from cells in TRI reagent (T9424, Sigma-Aldrich), digested with DNase I, and converted to cDNA using iScript gDNA Clear cDNA Synthesis Kit (172-5034, Bio-Rad Laboratories). To check whether or not target genes were amplified from contaminated genomic DNA, negative control without reverse transcriptase was included. Mouse hypothalamic cDNA was used as a positive control. PCR was performed for 40 cycles at 95 °C for 3 s and 60 °C for 30 s. PCR products along with a 50 bp DNA ladder (10416014, Thermo Fisher Scientific) were separated on 3% agarose gel in 1X TAE and visualized under UV light.

To measure expression levels of mRNA, total RNA was extracted from cells in TRI reagent and cDNA was prepared using Superscript IV (18090010, Thermo Fisher Scientific) or iScript Reverse Transcription Supermix (170-8840, Bio-Rad Laboratories). Expression levels of mRNA were measured by real-time PCR using the ABI 7500 Fast thermal cycler (Applied Biosystems, Foster City, CA) as described previously [61]. All primer pairs (Table 2) were designed using Primer Express software (Version 3.0, Applied Biosystems). Relative mRNA levels were determined using the ΔΔCt method by normalizing to cyclophilin or hypoxanthine-guanine phosphoribosyltransferase (*Hprt*) mRNA levels and were expressed as means (% of the control group) ± standard error of the mean (S.E.M.). All experiments were performed in triplicates and the coefficient of variation was less than 5% for each triplicate.

### 4.5. Statistical Analysis

Data are presented as means ± S.E.M. Outliers were identified by Discordance test and omitted from the analysis. Comparisons between two groups were done using Student’s *t*-test (parametric) or the Wilcoxon test (non-parametric). In the fructose treatment and 2,5-AHM treatment studies, data were analyzed by one-way ANOVA followed by the Turkey–Kramer post hoc test. In all cases, differences were taken to be significant if *p*-values were below 0.05.

## 5. Conclusions

The present study demonstrated that elevated glucose concentration leads to an increase in the level of microglial activation markers and the fructose transporter *Slc2a5* mRNA in cultured microglial cells and glucose-induced changes in pro-inflammatory genes expression is partially attenuated in microglia treated with the GLUT5 inhibitor. These findings support the hypothesis that nutrients affect the microglial activity and regulate the expression of glucose transporter genes. We propose that local glucose availability may affect the activity of microglial cells via both GLUT5-dependent and -independent carbohydrate sensing mechanisms. We also suggest that future research to focus on understanding the roles and regulation of microglial carbohydrate sensing and its association with the pathogenesis of obesity.

## Figures and Tables

**Table 1:** Effect of LPS on pro-inflammatory cytokine and *Slc2a5* mRNA expression in SIM-A9 cells.

Gene	Control	LPS (2.5 ng/mL)	*p* Value *
*Il1a*	100.0 ± 2.2%	308.9 ± 14.9%	<0.0001
*Il1b*	100.0 ± 5.9%	247,149.5 ± 12,442.2%	<0.0001
*Slc2a5*	100.0 ± 2.3%	154.1 ± 17.2%	<0.05

Values in control group were set to 100%. Data are means S.E.M. (*n* = 3–4/group). *: *p* values are obtained by Student’s *t*-test.

**Table 2:** Primer sequences used for real-time PCR.

Gene	Accession No.	Direction	Sequences	Exon
*Slc2a1*	NM_011400	Forward	5′-GGATCACTGCAGTTCGGCTAT-3′	2
		Reverse	5′-CGTAGCGGTGGTTCCATGTT-3′	3
*Slc2a2*	NM_031197	Forward	5′-CATTGCTGGACGAAGTGTATCAG-3′	4
		Reverse	5′-GGAGCGATCTCTCCAATGTACAT-3′	5
*Slc2a5*	NM_019741	Forward	5′-AAGCGACGACGTCCAGTATGT-3′	10
		Reverse	5′-GAATCGCCGTCCCCAAAG-3′	11
*Il1α*	NM_010554	Forward	5′-GCCCGTGTTGCTGAAGGA-3′	6–7
		Reverse	5′-TGGATAAGCAGCTGATGTGAAGTAGT-3′	7
*Il1β*	NM_008361	Forward	5′-TTGACGGACCCCAAAAGATG-3′	3
		Reverse	5′-TGCTGCTGCGAGATTTGAAG-3′	4
*Cyclophilin*	X52803	Forward	5′-AAGCATACAGGTCCTGGCATCT-3′	4
		Reverse	5′-TGCCATCCAGCCATTCAGT-3′	4–5
*Hprt*	NM_013556	Forward	5′-AGTCCCAGCGTCGTGATTAG-3′	1–2
		Reverse	5′-TGATGGCCTCCCATCTCCTT-3′	3

## Data Availability

The data presented in this study are available within the article or on request from the corresponding author.

## Supplementary Materials

The following are available online at https://www.mdpi.com/article/10.3390/ijms222312668/s1.
