# Supplementary material for: Regulation of the Fructose Transporter Gene Slc2a5 Expression by Glucose in Cultured Microglial Cells
Source: Int J Mol Sci. 2021 Nov 23;22(23):12668. doi: 10.3390/ijms222312668 (PMC8657830; doi:10.3390/ijms222312668)
Supplement: Supplementary file 1 [file ijms-22-12668-s001.zip › ijms-1467222-supplementary.pdf]

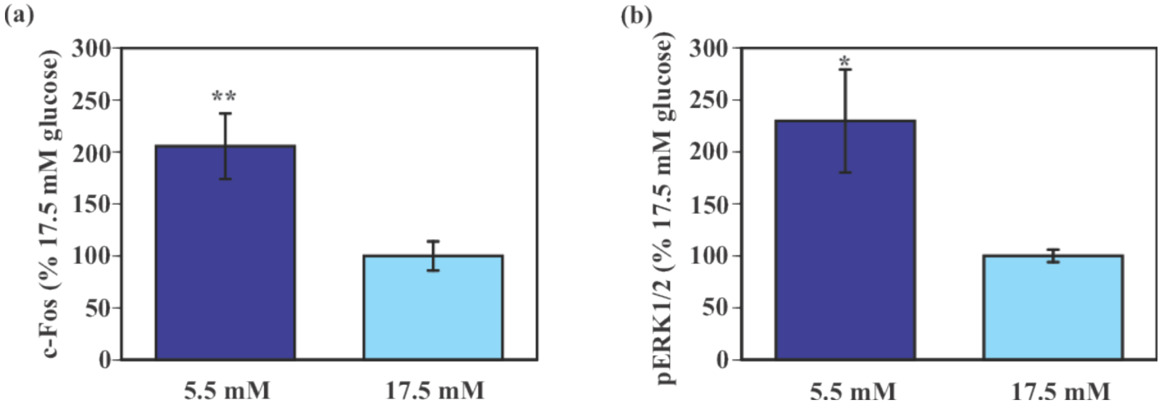

Figure S1. Low glucose concentration increases expression of microglia activation markers in murine microglial SIM-A9 cells. Cells were incubated in a culture medium containing either 17.5 mM or 5.5 mM glucose for 40 min. Levels of c-Fos **(a)** and pERK1/2 **(b)** were measured by Western blot analysis and were normalized to total protein from stain-free blot or total ERK1/2, respectively. Values in the control group (17.5 mM glucose) were set to 100%. Data are means ± S.E.M. (n = 10-12/group). *: $P < 0.05$, **: $P < 0.01$ by Student's *t*-test.
